# Complexity of the Therapeutic Regimen in Older Adults with Cancer: Associated Factors

**DOI:** 10.3390/ph17111541

**Published:** 2024-11-16

**Authors:** Rita F. Oliveira, Ana I. Oliveira, Agostinho Cruz, Oscar Ribeiro, Vera Afreixo, Francisco Pimentel

**Affiliations:** 1 REQUIMTE/LAQV, ESS, Polytechnic of Porto, Rua Dr. António Bernardino de Almeida 400, 4200-072 Porto, Portugal; aio@ess.ipp.pt (A.I.O.); agostinhocruz@ess.ipp.pt (A.C.); 2 Center for Health Technology and Services Research at the Associate Laboratory RISE—Health Research Network (CINTESIS@RISE), Department of Education and Psychology, University of Aveiro (UA), 3810-193 Aveiro, Portugal; oribeiro@ua.pt; 3 Center for Research and Development in Mathematics and Applications (CIDMA), Department of Mathematics, University of Aveiro (UA), 3810-193 Aveiro, Portugal; vera@ua.pt; 4 BlueClinical, 4460-439 Matosinhos, Portugal; fpimentel@blueclinical.pt

**Keywords:** Medication Regimen Complexity Index, MRCI, older adults, aging, cancer, polypharmacy

## Abstract

Background/Objectives: Population aging is a worldwide phenomenon and is often associated with multimorbidity and polypharmacy. Complex medication regimens are common among older adults and contribute to the occurrence of harmful health outcomes. Age is one of the main risk factors for cancer. This study aimed to determine and characterize the therapeutic complexity in older patients with cancer, and analyze the factors associated with high complexity and the impact of the oncological context. Methods: A cross-sectional study with patients aged ≥65 years with cancer was conducted in three hospitals in northern Portugal. Data collection was obtained using self-reports. The medication regimen complexity was assessed using the Medication Regimen Complexity Index (MRCI). Descriptive and association statistical analysis were performed. Logistic, linear, simple and multiple regression analysis were conducted, with and without automatic variable selection. Results: A total of 552 patients were included (median age, 71; IQR, 68–76). The mean MRCI before the oncological context was 18.67 (SD 12.60) and 27.39 (SD 16.67) after the oncological context, presenting a statistically significant difference in the values obtained (*p* < 0.001). An elevated complexity was significantly associated with polypharmacy, chronic diseases and with the administration of high-risk medications (*p* < 0.05). High MRCI values showed a relationship with the occurrence of potential drug interactions (*p* < 0.001). There was no relationship with the existence of cardiac risk comorbidity. Conclusions: This study demonstrated the existence of high therapeutic complexity in older patients with cancer, suggesting the need for intervention to prevent medication-related problems in this vulnerable population.

## 1. Introduction

Population aging is a global phenomenon. According to the World Health Organization (WHO), the number of older persons (≥65 years) is expected to reach 1.5 billion by 2050, representing approximately 16% of the population worldwide [1].

The high prevalence of chronic diseases in older adults [2] predisposes this population to the occurrence of polypharmacy contexts (use of five or more medications) and they have more complex medication regimens [3,4,5,6,7]. Due to the frequent presence of reduced manual dexterity and cognitive and sensory impairment they may face greater difficulty in managing their medication, making them more vulnerable to medication errors and medication-related problems [8,9].

Age is one of the main cancer risk factors due to biological changes associated with the aging process [10,11,12,13,14]. In the last few years there has been an increase in cancer incidence in many countries, which is primarily ascribed to a significant increase in the senior population. It is predicted that by 2040, 47% of all new cancer diagnoses will be in adults aged ≥70 years [11]. Older adults with cancer have a high comorbidities burden and polypharmacy is also common [15]. These patients have an additional medication burden because, in addition to cancer treatment, they are often administered medications to treat chronic diseases and supplementary supportive care medications. In being more vulnerable to adverse drug effects, geriatric patients with cancer undergoing chemotherapy tend to be more exposed to the risks of drug-related problems [16].

Therapeutic complexity may arise as a result of the number of medications, but other factors such as the administration of different dosage forms, multiple daily dosages and additional administration instructions must also be considered [8,9,17,18,19,20,21]. Medication complexity has been linked to negative health outcomes [22,23,24] such as non-adherence to medication [24,25,26], adverse drug reactions, drug interactions, hospitalizations [24,27], increased use of resources, increased cost of healthcare, decreased quality of life [28] and a decline in functional status and mortality [28,29,30]. In order to reduce the negative aspects identified, it is imperative to thrive in the simplification of medication regimens. Interventions that can reduce the complexity of medication and improve the patient’s quality of life and functional status are of great importance for older adults with cancer.

Different methods have been used to quantify the complexity of medication regimens. The Medication Regimen Complexity Index (MRCI), developed by George et al. [31], is the most used, reliable and validated tool for this purpose, having already been translated and validated into several languages [32,33,34,35,36] and applied in different contexts [37,38,39,40]. The MRCI is a tool that quantifies medication regimen complexity beyond the number of medications to include weighted scores for the types of dosage forms prescribed, dosing frequency and additional administration instructions, for each medication administered [31,41,42]. The MRCI allows a comprehensive and complete assessment and evaluation of a patient’s medication therapy regimen, allowing the identification of patients for intervention who require medication management [42,43]. The medication review has been shown to be an essential service to ensure medication safety in older patients with cancer, by preventing medication errors, identifying drug–drug interactions, adjusting chemotherapy doses and initiating deprescribing [44].

Complex medication regimens and the existence of polypharmacy make these patients more susceptible to the occurrence of medication errors and drug interactions, with particularly serious consequences in patients taking high-risk medications (e.g., warfarin, opioids, insulin) [38,45,46]. Likewise, careful attention should be given to patients with cardiovascular comorbidities and/or diabetes, which are prone to decompensate during anticancer treatment and often have been prescribed multiple drugs [21,43,44,47].

The present study aimed to determine and characterize therapeutic complexity in older patients diagnosed with cancer, and analyze the factors associated with high complexity and the impact of the oncological context on the complexity of the registered therapeutic regimen. This study also aimed to relate the MRCI to the existence of polypharmacy, potential drug interactions, administration of high-risk medications and the existence of comorbidities with cardiac risk.

## 2. Results

A total of 552 patients were included in this study, of which 308 were male (55.69%). The median age was 71 years (Interquartile Range (IQR), 68–76), with 8.88% of the patients being older than 80 years. The mean age was 71.88 years (SD 5.04). Regarding chronic diseases, 88.41% of the sample (N = 488) had at least one chronic disease and 60.14% (N = 332) presented more than two. Other common non-cancer diagnoses included hypertension (53.99%), dyslipidemia (38.95%) and diabetes mellitus (22.64%), in which 66.49% of the patients had at least one of the diseases (N = 367). The most common cancer types were “digestive system tumors” (36.23%), “lung, pleural, and thymic tumors” and “breast tumors” (both with 15.94%). The baseline characteristics of the sample are summarized in Table 1.

Regarding the medication administered, the prevalence of polypharmacy was 49.01% (N = 271) and a total of 266 (48.19%) patients took high-risk medications. Potential drug–drug interactions (DDIs) were identified in 76.45% of the patients (N = 422) and severe drug interactions (SDIs) were reported in 56.16% of patients (N = 310). The mean MRCI of all patients before the oncological context was 18.67 (SD 12.60), and 27.39 (SD 16.67) after the oncological context. The MRCI section with a higher mean was additional instructions followed by dosing frequency (Table 2).

Considering the patients’ oncological context, in the simple analysis the high total MRCI value was significantly associated with the existence of polypharmacy, excessive polypharmacy, chronic diseases, existence of comorbidities with cardiac risk (hypertension, dyslipidemia and diabetes mellitus) and the administration of high-risk medications (*p* < 0.001). There was no statistically significant relationship between total MRCI values and gender or age (*p* > 0.05). However, in a subsequent analysis, performing a multiple linear regression and analyzing all the variables, with and without automatic variable selection, only a significant association was observed between the total MRCI values with the existence of polypharmacy, excessive polypharmacy, chronic diseases and the administration of high-risk medications (*p* < 0.05) (Table 3).

MRCI values showed a statistically significant association with the occurrence of potential DDIs and SDIs (*p* < 0.001) (Table 4). The higher the MRCI values, the higher the number of DDIs and SDIs.

**Figure 1:**
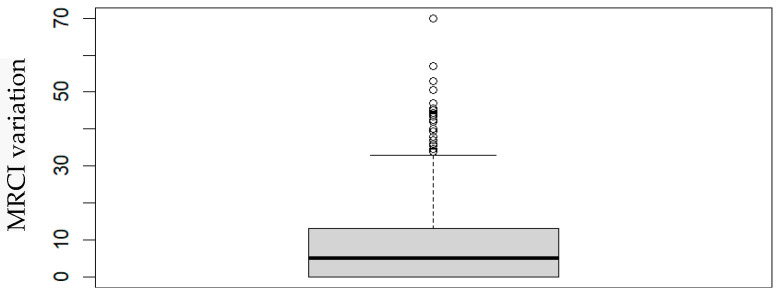
Boxplot of the MRCI variation at the two contexts.

In order to analyze the impact of the oncological context on the registered MRCI, we compared the final MRCI (which includes the medication administered in the oncological context) with the initial MRCI (considering only the medication before the oncological context). Comparing the MRCI before and after the oncological context, it was possible to observe a statistically significant difference between them (sign test; *p* < 2.2 × 10^−16^), which is higher considering the patients’ oncological context (Figure 1).

Carrying out a multiple logistic regression analysis, considering all variables, with and without automatic variable selection, it was found that the observed difference can be justified by polypharmacy, excessive polypharmacy and chronic diseases (*p* < 0.05), which appear to have an impact on MRCI values, and is higher when considering the patients’ oncological context. The previous presence of hypertension or dyslipidemia seems to have a smaller impact on the MRCI variation in both contexts. The data presented from the multiple analysis and AIC are coherent (Table 5).

## 3. Discussion

Our study aimed to quantify the complexity of the medication regimen using the MRCI tool and analyze associated factors in a sample of older patients with cancer. The results demonstrate the impact of the oncological context on the MRCI values obtained, observing a statistically significant difference when comparing the MRCI before and after the beginning of oncological treatment (*p* < 2.2× 10^−16^). The variables influencing the recorded difference were the existence of polypharmacy, excessive polypharmacy and chronic diseases (*p* < 0.05). The results can be justified by the necessity of administering more medications, including supportive ones. These findings can also be explained by the oncological context, which often involves the use of medications with more complex instructions for the treatment of various symptoms and/or associated complications.

The complexity of the therapeutic regimen was high (mean MRCI = 27.39), with values similar to those obtained in previous studies [48,49]. Different results were found in prior research that presented lower values [9,50,51,52,53]. It is important to note that to calculate the MRCI, and specifically regarding additional instructions for medication use, some studies assume that patients administered their medications according to the standard instructions for use described in key reference texts. Our study considers the user’s report regarding the effective use of the medication, which may justify the differences recorded.

A significant association was observed between the total MRCI values and the existence of polypharmacy, excessive polypharmacy, chronic diseases and the administration of high-risk medications (*p* < 0.05). The results are in line with previous studies that investigated the MRCI in older patients with multiple comorbidities and polypharmacy and who, consequently, present a greater complexity of the medication regimen [4,41,53,54,55].

It was also possible to observe that high MRCI values had a statistically significant relationship with the occurrence of potential DDIs and SDIs (*p* < 0.001). High MRCI values can, therefore, translate into greater patient exposure to the occurrence of drug interactions, which can compromise the effectiveness of treatments and jeopardize the patients’ safety [56]. There was no isolated, statistically significant relationship between the previous existence of diseases and cardiovascular risk (hypertension, dyslipidemia and diabetes mellitus) (*p* > 0.05). However, studies have recorded high MRCI values in patients with these pathologies. With the increasing prevalence of these diseases in the older cancer population, and the associated high treatment costs, simplification of medication regimens in these patients may be important to achieve intended therapeutic goals [57,58].

Although polypharmacy is identified as an important risk factor for the MRCI, Wimmer et al. (2016) states that the MRCI was a better overall predictor of mortality than polypharmacy, especially in patients ≤80 years old. Patients over 80 may have other non-drug risk factors for death and, as patients with a more limited life expectancy, may have more simplified therapeutic regimens [45]. Determining the MRCI is equally important because high values can lead to increased medication errors associated with the complexity of medication use instructions. A higher complexity regimen has also been associated with non-adherence to therapy [26,41], which, in turn, is a risk factor for therapeutic ineffectiveness, compromising the expected clinical result and ongoing treatment.

In the older population, medication administration is the main cause of preventable hospitalization due to the occurrence of adverse drug events (ADEs) [59], which may occur due to the complexity of the therapeutic regimen. An example of this is the administration of high-risk medications (e.g., anticoagulants, antiplatelets, oral hypoglycemic, insulins or opioid medications) due to a potentially variable dosage, the need for injection and possible transdermal distribution. These medications are also associated with potentially fatal ADEs, such as bleeding, hypoglycemia, falls and fractures [45,50,59,60].

The results obtained in this study reinforce the importance of high-risk medications in the MRCI. For this reason, although not included independently in the MRCI tool, the administration of these medications appears to be a relevant aspect to consider in determining the MRCI.

Because they are associated with ADEs and other complications, determining the MRCI is particularly important in severe and/or more demanding clinical contexts that require more complex medication regimens, such as chronic obstructive pulmonary disease, diabetes, patients with chronic kidney disease and diseases characterized by the use of medications with complex instructions for the treatment of comorbidities and complications associated with the disease itself or the treatment implemented, as in the case of cancer patients [45,61,62].

When we compare our results with MRCI studies in other disease contexts, we see that older adults with cancer have greater medication regimen complexity than patients with heart failure, heart transplant, depression, HIV, diabetes and hypertension [43,48,53,57,63,64]. Cancer patients are among the highest-risk patients and were most likely to have highly complex regimens. Many of these patients simultaneously have other chronic diseases, which aggravates the risk of the MRCI, which can translate into negative clinical outcomes.

In future studies, it is important to evaluate the impact of therapeutic complexity on hospital readmission and hospitalization in this group of patients. Previous studies conducted in other clinical settings found that medication regimen complexity was associated with hospital admissions and readmissions [65,66]. In some of these studies, the medication regimen complexity was not identified as a better predictor of hospitalization than the number of medications [27,29,50,61,67,68]. Chang et al. (2017), in their study, suggest that hospitalization appears to be associated with increased medication complexity and the overall number of medications prescribed [52]. The two parameters are considered good indicators of older patients’ risk of hospitalization [50].

Although studies suggest that the number of medications and the MRCI have a great impact, other factors may also be related to hospital readmission and hospitalizations, such as the patient’s own characteristics, existence of comorbidities, previous hospitalizations, length of stay, complexity of the pathological context and specific characteristics of certain medication. Even so, the complexity of the therapeutic regimen has a great impact on hospital readmission, presenting an important clinical implication, as, unlike simple medication counting, this tool has different parameters that can guide during the medication review [61]. The medication review has proven to be an essential task to ensure the safety of older cancer patients, preventing medication errors, identifying drug interactions, adjusting doses and initiating deprescription [44]. Future studies may include evaluating the use of the MRCI in clinical trial protocols for new chemotherapeutic agents, in order to optimize the therapeutic regimens implemented, ensuring continuity of treatments and avoiding medication-related problems. It is equally important to use the MRCI tool across specific cancer types and stages. This may allow us to obtain more precise conclusions and guide clinical practices in more specific contexts.

The MRCI is therefore a useful tool for identifying patients who may benefit from medication therapy management intervention [42]. Metz et al. (2014) stated that the use of the MRCI provided a better perspective of which patients might be at greater risk for failing to achieve desired outcomes [64]. This tool therefore does not simply assess the number of medications a patient is taking: it also assesses points (indicating greater complexity) for the formulation (e.g., that require special devices, such as inhalers or injections, that are more complex than a tablet), dosing frequency (e.g., more times per day is more complex) and any additional directions the patient needs to follow (e.g., take at a specific time of day; take 1 h before meals; take medication on an empty stomach; and the need to divide tablets) [31,32].

Multiple formulations, several dosing frequencies and additional instructions likely complicate any patient’s ability to maintain proper and consistent medication administration practices. Case of older adults may be even more demanding due to the limitations in vision, hearing, dexterity or memory, with impaired cognition and polypharmacy [17,24,53,62]. Similar to previous studies, in our results the dosing frequency and additional instructions are the two parameters that most contributed to the overall MRCI score. These parameters should be considered by health professionals in the medication therapy management intervention.

Our results suggest that medication regimens should be reviewed for possible complexity reductions, such as removing unnecessary medications (reducing the number of medications administered) and/or simplifying dosing regimens and instructions for medication administration. Reducing dosing frequency has been identified as the intervention with the greatest potential to simplify the therapeutic regimen, particularly through the use of long-acting medications [4,27,69]. This may be of particular relevance in older patients presenting cognitive deficits and without support in managing their medication [61]. As it is not possible to safely reduce complexity, because all prescribed medications are necessary, knowledge of this is essential as it allows the identification and reduction of risks associated with the high medication regimen complexity [42].

Although determining the MRCI is not as immediate and easy to implement in routine clinical practice compared to determining the number of medications, studies show that MRCI scores can be automatically calculated and integrated into electronic health records in a way that it can assist clinical decision making [43,50,61,70]. It is therefore necessary to simplify and automatize the MRCI application to become a practical, fast, feasible and easy-to-use tool when caring for patients and managing drug therapy in primary care, in community pharmacies and in a hospital context. The MRCI can be used as a tool in clinical and pharmaceutical practice to identify patients whose therapeutic regimens are highly complex and who can benefit from an intervention. The identification of factors associated with high therapeutic complexity is useful in order to direct interventions to be implemented, and thus prevent unwanted clinical outcomes such as ADEs and hospitalizations [42,71].

### Strengths and Limitations

To our knowledge, this is the first study carried out in older people in an oncological context that investigated therapeutic regimen complexity and its relationship with polypharmacy, drug interactions, the existence of chronic diseases, the presence of cardiovascular risk diseases and the administration of high-risk medications common in the geriatric population. The data were collected by trained staff and the medication regimen complexity was measured using a validated measure. It is important to acknowledge that despite the relevance of the reported data, our results may not be generalizable to other older populations due to the median age of the sample (71 years) as it is not a very aged sample in relation to average life expectancy. In addition, there may be other variables not considered, which may alter the results obtained. Furthermore, there are differences in the structure and availability of health and social services in relation to other countries. Also, although we adjusted our analyses for clinically important variables, as with all observational studies, the possibility of residual confounding cannot be excluded. Another potential limitation is that the determination of the MRCI occurs only at one point in time; however, patients receive care from multiple healthcare professionals during their treatment period, which may result in changes in medication and consequently in its complexity regimen. In other words, our analysis did not consider changes in medication regimens during the treatment period. In this study, the suitability of medications administered by patients was not analyzed. Data were collected through self-report, which may have resulted in inaccuracies and/or omissions of administered medications. Still, questioning patients about their medication use, rather than analyzing data on prescribed or dispensed medications, provided potentially more accurate information on actual medication use.

## 4. Materials and Methods

### 4.1. Study Setting, Participants and Eligibility

This is a cross-sectional study conducted at three hospitals in Porto, in northern Portugal, during a period of 16 months. It included 552 participants. Patients were flagged and invited to participate in this study by the nursing team while undergoing their chemotherapy treatment, respecting this study’s inclusion criteria: older adults with a diagnosis of cancer, aged 65 or over, with no cognitive impairment. Cognitive status was assessed using the six-item Cognitive Impairment Test (6 CIT) [72]. The exclusion criteria were not mastering the Portuguese language or not being responsible for managing one’s own medication. Data collection was carried out by the research team through direct contact with patients. Patients with incomplete data were excluded from the analysis. A non-probabilistic sampling for convenience was performed, in which the sample size was calculated using EpiInfo™^®^ (Version 7.1.5/2015). This study was carried out after approval by the Health Ethics Committees of the three hospital institutions where this study took place and written informed consent to participate was obtained for each participant.

### 4.2. Data Collection

Data collection was conducted using a structured questionnaire applied to all participants. The collected data included standard demographic information (age and sex), medical conditions, identifying the type of cancer and the existence of other chronic diseases, including diseases with cardiovascular risk (diabetes mellitus, hypertension and dyslipidemia), and a detailed list of all medications administered. Information about medication use was obtained using self-reports. Information on the pharmaceutical form, therapeutic regimen and administration precautions was collected. High-risk medications were identified according to the following categories: anticoagulants, antiplatelet agents, insulin, oral hypoglycemic agents, opioids and antiarrhythmic drugs [59,73]. The oncological context of the patients was coded by the International Statistical Classification of Diseases and Related Health Problems 10th Revision (ICD-10)—WHO (Version 2019).

### 4.3. Outcome Measurements

In this study, polypharmacy was defined as the use of five or more medications. The use of ten or more medications was labeled excessive polypharmacy [73,74,75,76,77]. Potential drug–drug interactions (DDIs) were assessed using the Micromedex^®^ (electronic) [78]. The most valued and clinically relevant DDIs were severe drug interactions (SDIs), which included major and contraindicated interactions. The Micromedex^®^ solutions database has been used in other oncology drug interaction studies [79,80].

### 4.4. Assessment of Medication Regimen Complexity

The medication regimen complexity was assessed using the MRCI. Originally developed and validated by George et al. (2004), the MRCI is a standardized tool, considered valid and reliable for measuring the complexity of medication regimens [31,32,42]. The MRCI is a 65-item instrument that can be used to quantify medication regimen complexity, and presents three sections: (A) dosage form (32 items) (tablet/capsule, paste, injectable, etc.); (B) dosing frequency (23 items) (once a day, twice a day, etc.); and (C) additional instructions for use (10 items) (take with food, crush/break the tablet, alternating dosage, etc.). The MRCI is an open-ended instrument in which the total MRCI score is calculated by summing the scores from each section and where higher total MRCI scores represent more complex medication regimens [31,41,42].

### 4.5. Statistical Analysis

The data were summarized by location measures (mean, median, minimum and maximum) and dispersion measures (standard error and Interquartile Range, IQR). The variables under study presented a non-gaussian distribution. Quantitative variables were analyzed through the Wilcoxon–Mann–Whitney Test; qualitative variables were analyzed with Pearson’s chi-square test; and the association between two quantitative variables was evaluated with Spearman’s correlation test (and described by the corresponding correlation coefficient). Logistic, linear, simple and multiple regression analysis were conducted, with and without automatic variable selection. Automatic selection was carried out using the Akaike Information Criterion (AIC). The association effect sizes were measured as the odds ratio (OR) or linear coefficient regression (beta). All statistical procedures and analysis were performed with R version 4.3.2. Statistical hypothesis tests with *p*-values less than 0.05 were considered significant. Confidence intervals are reported with a 95% confidence level.

## 5. Conclusions

This study demonstrated the existence of high therapeutic complexity in older patients with cancer, suggesting the need for intervention to prevent medication-related problems in this population. The complexity of drug treatment is known to be a risk factor for administration errors and therapeutical non-adherence, which can compromise the safety and effectiveness of the treatment and promote higher healthcare costs, hospital admissions and increased mortality. Older adults with cancer need a regular review and optimization of their prescriptions. The therapeutic review represents an opportunity to deprescribe and simplify medication regimens. Research is needed to better understand the impact of using multiple medications and the effect of medication optimization interventions on clinical outcomes in these patients.

## Figures and Tables

**Table 1:** Baseline characteristics of enrolled patients (N = 552).

Variable	n	%
Age, median (IQR), years	71	(68–76)
Age, mean (SD)	71.88	(5.04)
65–79	503	91.12%
>80	49	8.88%
Sex		
Male	308	55.69%
Female	244	44.31%
Comorbidities		
No	64	11.59%
Yes	488	88.41%
≥2	332	60.14%
Comorbidities/Diseases		
Heart	353	63.95%
Endocrine	143	25.91%
Osteoarticular	109	19.75%
Visual	85	15.40%
Digestive	78	14.13%
Respiratory	65	11.78%
Neurological	56	10.14%
Other	240	43.48%
Comorbidities with cardiac risk (at least one)	367	66.49%
Diabetes mellitus	125	22.64%
Hypertension	298	53.99%
Dyslipidemia	215	38.95%
Cancer type (ICD-10)		
Malignant neoplasms of digestive organs	200	36.23%
Malignant neoplasms of respiratory and intrathoracic organs	88	15.94%
Malignant neoplasm of breast	88	15.94%
Malignant neoplasms of male genital organs and urinary tract	60	10.87%
Other	116	21.01%

Abbreviations: IQR, Interquartile Range; SD, standard deviation.

**Table 2:** Medications and MRCI.

Variable	Descriptive Statistics/Frequency	
	n	%
Medications		
0–4	239	43.3%
≥5	271	49.01%
≥10	42	7.61%
High-risk medication	266	48.19%
Patients exposed to DDIs	422	76.45%
Patients exposed to SDIs	310	56.16%
MRCI Initial (before the oncological context)		
Mean, median (SD, IQR)	18.67, 16.00 (12.60, 9.38–24.63)	
MRCI Final (after the oncological context)		
Mean, median (SD, IQR)	27.39, 23.75 (16.67, 16.00–38.00)	
MRCI—Sections (after the oncological context)		
Section A—Dosage Form, mean (SD)	2.07 (2.02)	
Section B—Dosing Frequency, mean (SD)	6.48 (4.42)	
Section C—Additional Instructions, mean (SD)	18.8 (11.7)	

Abbreviations: DDIs, drug–drug interactions; SDIs, severe drug interactions; MRCI, Medication Regimen Complexity Index; SD, standard deviation; IQR, Interquartile Range.

**Table 3:** Factors associated with the MRCI (linear regression for the MRCI outcome).

	Simple			Multiple			Best AIC		
Characteristic	Beta	95% CI	*p*-Value	Beta	95% CI	*p*-Value	Beta	95% CI1	*p*-Value
Age	0.10	−0.17, 0.36	0.476	−0.05	−0.18, 0.08	0.412			
Sex	−1.3	−4.1, 1.6	0.382	0.41	−1.2, 2.1	0.621			
Polypharmacy	26	24, 28	<0.001	21	19, 22	<0.001	21	20, 23	<0.001
Excessive polypharmacy	39	35, 43	<0.001	27	24, 29	<0.001	27	24, 29	<0.001
Chronic diseases	12	7.9, 16	<0.001	3.5	1.2, 5.8	0.003	3.6	1.5, 5.7	<0.001
Hypertension	8.3	5.6, 11	<0.001	0.28	−1.3, 1.8	0.723			
Dyslipidemia	8.2	5.4, 11	<0.001	−0.32	−1.9, 1.2	0.686			
Diabetes mellitus	12	8.4, 15	<0.001	1.1	−0.63, 2.8	0.214			
High-risk medications	13	10, 16	<0.001	3.2	1.0, 5.3	0.004	2.9	1.1, 4.8	0.002

Abbreviations: CI, confidence interval; AIC, Akaike Information Criterion.

**Table 4:** Association between the MRCI and the occurrence of drug interactions (linear and logistic regression).

	DDIs (Quantitative)			SDIs (Binary)		
Characteristic	Beta	95% CI	*p*-Value	OR	95% CI	*p*-Value
MRCI Final	0.11	0.09, 0.13	<0.001	1.05	1.04, 1.07	<0.001

Abbreviations: DDIs, drug–drug interactions; SDIs, severe drug interactions; MRCI, Medication Regimen Complexity Index; CI, confidence interval; OR, odds ratio.

**Table 5:** Factors associated with the MRCI at the two contexts (before and after the oncological context).

	Simple			Multiple			Best AIC		
Characteristic	Beta	95% CI	*p*-Value	Beta	95% CI	*p*-Value	Beta	95% CI	*p*-Value
Age	−0.05	−0.24, 0.13	0.562	−0.11	−0.26, 0.05	0.179			
Sex	−1.9	−3.8, 0.06	0.057	−1.7	−3.6, 0.25	0.087	−1.4	−2.9, 0.19	0.085
Polypharmacy	11	9.8, 13	<0.001	10	8.2, 12	<0.001	10.0	8.3, 12	<0.001
Excessive polypharmacy	18	15, 22	<0.001	13	10, 16	<0.001	13	10, 16	<0.001
Chronic diseases	5.6	2.6, 8.6	<0.001	3.7	1.0, 6.4	0.007	3.5	0.88, 6.2	0.009
Hypertension	1.2	−0.79, 3.1	0.243	−2.2	−4.1, −0.37	0.019	−2.3	−4.1, −0.52	0.011
Dyslipidemia	1.3	−0.65, 3.2	0.195	−1.8	−3.5, −0.02	0.047	−2.0	−3.7, −0.33	0.019
Diabetes mellitus	3.4	1.2, 5.7	0.003	−0.46	−2.4, 1.5	0.646			

Abbreviations: CI = confidence interval; AIC, Akaike Information Criterion.

## Data Availability

Data are contained within this article.
